# The Effects of Thirdhand Vape Residue from Nicotine and Non-Nicotine Vapes on Cells: A Systematic Review

**DOI:** 10.3390/ijerph22040465

**Published:** 2025-03-21

**Authors:** Jazzlin Marie Adele Stracci, Alyssa Priyanka Ganesan, Prescious Grace Pitogo, Sheree Margaret Smith

**Affiliations:** 1 School of Biomedicine, Faculty of Health and Medical Sciences, University of Adelaide, 5005 Adelaide, Australia; alyssa.ganesan@student.adelaide.edu.au (A.P.G.); presciousgrace.pitogo@student.adelaide.edu.au (P.G.P.); 2 Adelaide Nursing School, Faculty of Health and Medical Sciences, University of Adelaide, 5005 Adelaide, Australia; sheree.smith@adelaide.edu.au

**Keywords:** thirdhand smoke, vapes, animal models, cells

## Abstract

Rationale: Vapes are increasingly popular, however, their clouds leave a residue on surfaces, referred to as thirdhand smoke. Recent studies have reported the detrimental cellular impacts of thirdhand smoke. However, research on thirdhand vape residue exposure is relatively new and understudied. Objectives: This review aims to evaluate the current literature associated with the impact of thirdhand vape residue from nicotine and non-nicotine vapes on cells, compared to unexposed controls. Methods: A systematic search was performed in PubMed (Medline), Embase, Web of Science, Scopus, and Google databases to identify relevant studies. Two independent reviewers screened articles using the inclusion criteria of controlled experimental studies on human and animal in vitro and in vivo models which investigate thirdhand vape residue as the exposure variable and cell concepts. Studies were assessed for bias through tools specialised for animal studies. Data were extracted and synthesised in accordance with PRISMA guidelines. Results: Of 139 articles retrieved, three are included in this review, focusing on mice cell models only, one of which investigates non-nicotine vapes. No studies on human cell models that fit the criteria were found. Mice were directly exposed to vape-infused materials from which their cells were extracted and evaluated, finding that exposure to thirdhand nicotine vape residue damages mice cells. The effects of non-nicotine vapes are inconclusive. Conclusions: Thirdhand nicotine vape residue contributes to changes in some cells in mouse models but not others. Data available to date provide no convincing evidence of likely significant harm to humans. Further investigation is warranted to confirm or deny this impression.

## 1. Introduction

### 1.1. Background

Vapes, or e-cigarettes, were initially introduced in 2008 and marketed as an alternative cessation tool for traditional cigarette smoking [1]. However, vapes, particularly those containing nicotine, are becoming widely normalised with their daily usage in 2022–2023 triple that in 2019 [2]. Contributing to the increase in vaping are the various pleasantly flavoured ‘e-liquids’, which have appealed to younger populations with nearly 50% daily vape usage in people aged 18–24 [3].

The e-liquids in nicotine and non-nicotine vapes contain polypropylene glycol (PG) and vegetable glycerine (VG), which during inhalation undergo thermal degradation and oxidise, forming toxic aldehydes [4,5]. The PG/VG ratio in vapes determines the vaping experience with high VG producing larger clouds of vapour and high PG producing stronger flavours [4]. Other toxic chemicals are also found in the flavour component of e-liquids, and react with PG and VG to form irritant acetal compounds which are highly cytotoxic to the respiratory tract [6]. Studies reveal these constituents may lead to increased production of reactive oxygen species, pro-inflammatory cytokines and fibroblasts, cellular death, and altered structure and function of respiratory cells [7,8,9].

These cellular effects of vaping are not only relevant to the consumer, but also to those indirectly exposed, through the inhalation of secondhand smoke [10] and thirdhand smoke, which is a relatively new concept in public health. Thirdhand smoke is the residual components and byproducts of smoke clouds that remain seeped into surfaces or re-emitted into gases even years after the smoking has occurred. This can be skin absorbed, inhaled, and ingested [10,11].

### 1.2. Rational

The cellular effects of firsthand smoke have been well documented. Current research on thirdhand smoke focuses more on traditional cigarettes [12], although some evidence does exist on dose responses to vapes, mainly in mouse models where prescribed timing is reported [13]. Due to the increasing popularity and use of vapes, it is vital to examine the effects of thirdhand vape residue exposure related to nicotine and non-nicotine vapes and its effects on cells. This is particularly important for vulnerable populations like children, who are more susceptible to the effects of thirdhand vape residue due to inquisitive behaviours, and thinner skin facilitating increased absorption [14].

### 1.3. Aims

To date, a review of the effect of thirdhand vape residue exposure has not been undertaken. Therefore, the aim of this review is to evaluate the literature investigating the impacts of thirdhand vape residue from nicotine and non-nicotine vapes on cells. This includes all cell types and concepts, including type and extent of changes in function and histology compared to normal unexposed cells.

## 2. Materials and Methods

### 2.1. Study Inclusion and Exclusion Criteria

This review focused on controlled experimental studies conducted on in vitro and in vivo models of both animals and humans, with no limitations on species, sex, or group numbers. Non-English studies and non-original research including review articles, reports, letters to the editor, and books were excluded. The studies were selected if thirdhand nicotine and non-nicotine vape residue was the exposure variable seen through title and abstract screening, as well as observed associations between this and the effect on cells seen at full-text screening. Eligible intervention administrations included environmental exposure of cells through infused materials or surfaces, or exposure to vape aerosol deposits at levels typically experienced thirdhand. The main outcome to be observed was the measures of the association between exposure to thirdhand vape residue and the cellular outcomes of such compared to a control. Any studies on general health effects were excluded.

### 2.2. Literature Search Strategy

Between inception from June to October 2024, we carried out a search of English published articles in PubMed (Medline), Embase, Web of Science, and Scopus databases on the domain of thirdhand nicotine and non-nicotine vape exposure and all associated cellular effects. Furthermore, a general Google search and forward and backward searches of reference lists of relevant study articles and systematic reviews were conducted to identify published grey literature on the topic.

In our search strategy, general terms such as ‘thirdhand’, AND ‘vaping’ AND ‘cell’ and analogues of such were searched within the title and abstract, as well as terms revolving around the concept of cells such as ‘cytology’ OR ‘DNA’ OR ‘epithelial’ in all fields to increase article numbers. The MeSH terms (mh) ‘vaping’ and ‘Electronic Nicotine Delivery Systems’ were also incorporated into the search to find intervention-specific articles. Boolean operators ‘AND’ and ‘OR’ are used in between terms to find more specific and accurate literature, as well as truncation of an asterisk “*” to broaden the literature. The search queries and results are shown in Table 1.

### 2.3. Data Screening and Extraction

The lead investigator independently screened the title and abstract and full text of data from databases in accordance with the inclusion criteria. We used Covidence for further screening of this data, where a second independent reviewer filtered the articles selected to be included or excluded in the systematic review. Any disagreements were resolved via group consensus, or the opinion of a final reviewer when consensus was not reached.

The lead investigator independently extracted the data from the text, figures, and graphs in accordance with PRISMA guidelines. Data were extracted on study design and cell type, specifying whether they were animal- or human-derived, or cells in commercially available cell lines. In each study, the intervention of interest was extracted, including dose response to vape aerosol and deposits where applicable and prescribed timing of exposure. This included recording the concentrations and vape characteristics. The outcome was extracted as continuous data of mean and standard deviation (SD) or standard error of the mean (SEM) and analysed using one-way or two-way analysis of variance (ANOVA), using Tukey post hoc tests.

### 2.4. Risk and Reporting of Bias

**Figure 1:**
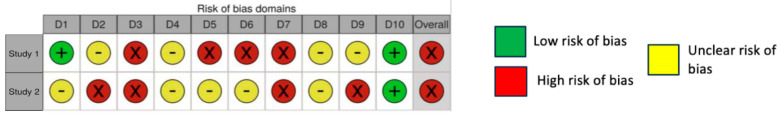
Example of use of SYRCLE (Systematic Review Centre for Laboratory Animal Experimentation) Risk of Bias Tool for Animal Studies, where studies are assessed for risk of bias according to various domains (D1–D10) [15]: D1: Random sequence generation (selection bias); D2: Baseline characteristics (selection bias); D3: Allocation concealment (selection bias); D4: Random housing (performance bias); D5: Blinding of participants and personnel (performance bias); D6: Random outcome assessment (detection bias); D7: Blinding of outcome assessment (detection bias); D8: Incomplete outcome data (attrition bias); D9: Selective reporting (reporting bias); D10: Other bias.

We assessed studies for their risk of bias using the guidelines recommended in the SYRCLE (Systematic Review Centre for Laboratory Animal Experimentation) Risk of Bias Tool [15] for Animal Studies, as shown in Figure 1.

**Figure 2:**
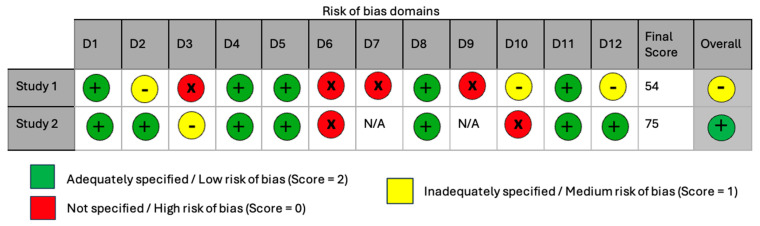
Example of use of QUIN Risk of Bias tool (Quality Assessment Tool For In Vitro Studies) for in vitro human cell studies, where studies are assessed for risk of bias according to various domains (D1–D12) [16]: D1: Clearly stated aims/objectives; D2: Detailed explanation of sample size calculation; D3: Detailed explanation of sampling technique; D4: Details of comparison group; D5: Detailed explanation of methodology; D6: Operator details; D7: Randomisation; D8: Method of measurement of outcome; D9: Outcome assessor details; D10: Blinding; D11: Statistical analysis; D12: Presentation of results; Final score: = (Total score × 100)/(2 × number of criteria applicable).

The QUIN risk of bias tool (Quality Assessment Tool For In Vitro Studies) [16] was considered for in vitro studies of human-derived cells when appropriate, as shown in Figure 2. Two independent reviewers assessed the risk of bias in each study using a traffic light marking system to assess for low, some, or high bias according to the different domains of the tool. The last reviewer also resolved discrepancies that arose in this process.

The lead investigator also checked that all studies had institutional ethical approval, no conflicts of interest, and acknowledged funding which may influence the results otherwise. Studies assessed to be at elevated risk of bias were highlighted according to the narrative synthesis.

### 2.5. Data Synthesis

A narrative synthesis of data was conducted in accordance with PRISMA guidelines for a systematic review, summarising findings from studies to formulate conclusions on the effects of thirdhand vape residue from nicotine and non-nicotine vapes on cells. The PRISMA guidelines served as a checklist during the data synthesis and were used to illustrate the flow of information and reporting of this systematic review. The data were analysed in subgroups distinguishing between nicotine and non-nicotine vape effects.

This systematic review was registered with Open Science Framework and can be accessed at: https://doi.org/10.17605/OSF.IO/5P2RY.

## 3. Results

### 3.1. Study Selection

**Figure 3:**
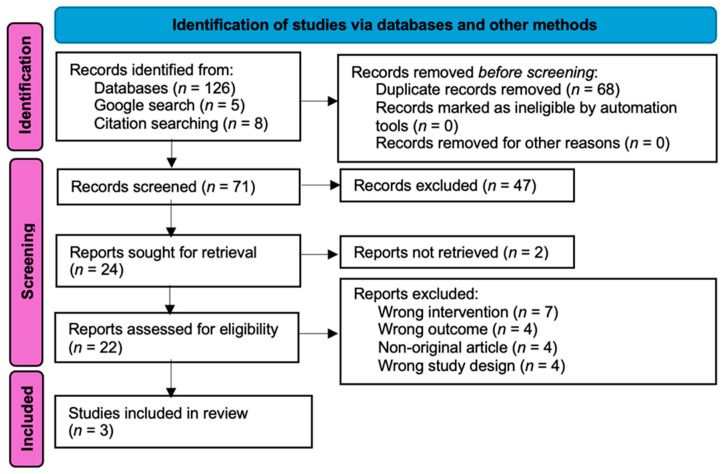
The PRISMA flow diagram of screening strategy and study selection [19].

A total of 139 articles were retrieved during the initial database searches, of which 71 unique articles were identified. Following the inclusion criteria, after title and abstract screening, 22 articles remained for full-text assessment. Of these, 19 articles were excluded due to wrong outcomes or no or limited cellular investigation or studies of general health effects (*n* = 4), wrong study design being uncontrolled or non-experimental (*n* = 4), non-original articles of systematic reviews (*n* = 3) or letters to the editor (*n* = 1), and wrong intervention of other exposure types like secondhand smoke (*n* = 3), exposure to cigarettes (*n* = 2), or thirdhand exposure to only the nicotine component of vapes in two studies by Olomu et al. [17] and Pozuelos et al. [18]. These studies were excluded after a final consensus because although they focused on the cellular outcomes of exposure of human cells to nicotine concentrations typically experienced in thirdhand vape contexts, they did not expose the cells to the other chemicals typically present in vapes. Therefore, the results of these studies are not entirely relevant to the aim of this review. Ultimately, a total of three articles on mice models were selected to be included in this systematic review (Figure 3).

### 3.2. Characteristics of Included Studies

The characteristics of the three included studies are summarised in Table 2. These included mouse cell models for the investigation of the impact of thirdhand vape residue. Of these three studies, two [20,21] were carried out in the United States of America and one [13] in Australia.

All three studies [13,20,21] focused on mice models. Following the inclusion criteria of this review, all selected studies adopted a controlled experimental design type [13,20,21]. Of these, one was specifically randomised experimental [13]. These experimental studies involved the assignment of mice to an experimental group exposed to vape smoke containing nicotine typical of thirdhand levels of vapes or a control group of clean air exposure. Thorpe et al. [13] differed having two experimental groups, exposed to vape smoke containing 18 mg nicotine or 0 mg nicotine. Sample sizes in the included studies ranged from 5 to 10 mice per group. Umphres et al. [20] placed 5–8 male and female mice, aged six weeks, in the experimental and control group. Thorpe et al. [13] randomly assigned 10 male mice, aged four weeks, into the two experimental groups, and the control group. Commodore et al. [21] allocated seven female and male mice, aged four or eight weeks, into the experimental group and six into the control group.

The cells examined in these studies included airway smooth muscle, epithelium and collagen cells (*n* = 1) [13], inflammatory cells (leukocytes, macrophages, neutrophils, eosinophils) in the blood (*n* = 1) [20], lung, brain, and liver tissue (*n* = 1) [21], and bronchoalveolar lavage fluid (BALF) (*n* = 1) [13]. In addition to cell analysis, fragments of cells called platelets were also examined in one study [20], as well as cell-secreted cytokine and chemokine proteins in the serum and BALF (*n* = 1) [21].

### 3.3. Vape Exposure and Outcome Measures of Included Studies

The experimental conditions of each study, detailing the exposure of mice cells to vapes and the outcome measures of this in terms of cellular and statistical analysis, are summarised in Table 3.

In all three studies [13,20,21], mice lived in cages furnished with vape-infused cotton fabrics or clean unexposed fabrics to compare the effects of thirdhand exposure on various cell types. These fabrics were placed in a vapor inhalation exposure system, and infused with vape smoke with 6 mg (*n* = 1) [21], 18 mg (*n* = 2) [13,20], or 0 mg (*n* = 1) [13] of nicotine. Thorpe et al. [13] were the only researchers to investigate non-nicotine vapes in their study. However, the flavours and ratios of PG/VG differed from a 50/50 PG/VG unflavoured vape in Commodore et al.’s [21] study, a 50/50 PG/VG tobacco flavoured vape in Thorpe et al.’s [13] study, and a 30/70 PG/VG menthol flavoured vape in Umphres et al. [20] study.

Umphres et al. [20] exposed the fabrics in their study to 400 puffs of e-cigarette vapour daily for seven days. These puffs lasted three seconds and were delivered at a 1 L/min airflow. The mice were exposed to the materials for four months before blood was extracted. Platelets in the blood were split into three groups all activated with 0.05 units/mL of thrombin or 1 µM of adenosine diphosphate platelet agonist. Using an aggregometer, one group was assessed for platelet aggregation, and another treated with 12.5 µL of luciferase substrate before activation to evaluate dense granule secretion response. The third group of platelets were washed and incubated with anti-mouse antibodies to assess flow cytometric outcomes using a cytometer.

In another study, Thorpe et al. [13] exposed cotton towels to 20 puffs, equal to 250 mL of 18 mg nicotine or 0 mg nicotine e-cigarette vapour. The mice in the control and experimental groups were exposed to the towels, which were replaced daily for four weeks. Mice were then euthanised, and BALF was extracted, processed, and stained and the histology of cells was analysed through imaging. Lungs were also removed and sectioned, by which mRNA was extracted from cells and analysed using PCR.

Finally, Commodore et al. [21] exposed cotton towels to 60 puffs of e-cigarette vapour for one hour. These puffs lasted for 3.3 s at an airflow volume of 70 mL/min. Mice were exposed to these towels for one hour daily for five days followed by a two-day break over a three-week period. A new towel was infused and used after each period of five days; 100 μL of serum and BALF were extracted from the mice analysed through multiplexing analysis of protein data and cell counts.

Data were analysed in the three studies as mean [13,20,21] as well as SEM (*n* = 1) [13] or SD (*n* = 2) [20,21]. One-way ANOVA (*n* = 3) [13,20,21] and two-way ANOVA (*n* = 1) [13] tests were used in these studies to compare the mean effects in experimental and control mice. A *p*-value < 0.05 is indicative of statistically significant results. *t*-tests were conducted in 2 studies [13,20] and calculated via Tukey’s post hoc tests (*n* = 1) [13] due to the small sample size, or a method not mentioned (*n* = 1) [20].

### 3.4. Risk of Bias of Included Studies

Assessing the risk of bias of these three studies [13,20,21] is vital to determine the quality of the stud, and the reliability and validity of the outcomes produced. The SYRCLE Risk of Bias tool was used in assessing these studies on mice cells summarised in Table 4. The QUIN Risk of Bias tool was not used as there were no studies included on human models. All three studies [13,20,21] received institutional ethical approval to conduct their experiments, had no conflicts of interest, and acknowledged funding indicating no influences on the results.

As shown in Table 4, concerns exist on possibly biased randomisation of mice to groups, concealed allocation to groups, and random selection of animals for assessment in all three studies [13,20,21] due to missing information. The baseline characteristics of the groups were similar and were not adjusted for by Commodore et al. [21] indicating high risk of bias. It is unknown whether mice were housed randomly or if investigators were unblinded but this likely had no effect on the results (*n* = 3) [13,20,21]. In the three studies [13,20,21], it is unknown whether the outcome assessor was blinded, however, lack of blinding is likely to affect the outcomes of results in one study [13] due to subjectivity and selectivity of outcome assessment. Two studies [20,21] included all animals in their analysis apart from one [13], however, it is unknown whether this was due to mice death. However, in the three studies [13,20,21], the results of all pre-specified analysis methods and statistical reports were included (D5). There was uncertainty in a number of studies due to excessive missing information to enable a complete assessment of the risk of bias in these studies. In future studies, research authors will be contacted for missing information.

### 3.5. Effect of Thirdhand Exposure to Nicotine Vape Residue

Due to the increasing popularity of nicotine vapes, investigating the cellular impacts of thirdhand vape residue from these is vital. The combined results of the three studies [13,20,21] examining inflammatory cells revealed that macrophages (*n* = 2) [13,21] and leukocytes were decreased non-significantly (*n* = 1) [13], and lymphocytes were decreased non-significantly in one study [20] and increased non-significantly in another [21]. Interestingly neutrophils were increased and eosinophils decreased in one study [21], with neutrophils decreased and eosinophils increased in another [13]. However, these results did not demonstrate significant changes. Commodore et al. [21] noted a statistically significant decrease in cytokine Il-7 levels (*p* = 0.010) in the serum and increased Il-13 levels (*p* = 0.017) in the BALF of experimental mice which activate and regulate these inflammatory cells. However, other proteins were viewed to be insignificantly affected.

Two [13,20] animal studies revealed evidence of hyperactivity of cells. This includes statistically significant increases in platelet aggregation (*p* < 0.01) and dense granule secretion of platelets (*p* < 0.05) related to increased levels of surface *p*-selectin (*p* < 0.0001), platelet integrin αIIbβa3 activation (*p* < 0.0001), and phosphatidylserine enhancement (*p* < 0.0001) as seen in Umphres et al.’s study [20]. Thorpe et al. [13] reported that exposure to thirdhand nicotine vape residue hyperactivated cells, resulting in minorly increased airway smooth muscle thickness in small and large airways, and significantly increased (*p* < 0.05) number of epithelial layers in small airways and epithelial thickness in large airways. This is combined with significantly increased (*p* < 0.05) airway collagen in small airways only due to increased lung col1α1 and col4α1 gene expression. This contributed to a statistically significant reduction in central resistance (*p* < 0.05), transpulmonary resistance (*p* < 0.01), tissue damping (*p* < 0.01), and tissue elastance (*p* < 0.01) following exposure, compared to controls. Interestingly, exposure to thirdhand nicotine vape residue resulted in significantly enlarged alveoli (*p* < 0.001) representing emphysema.

### 3.6. Effect of Thirdhand Exposure to Non-Nicotine Vape Residue

The majority of evidence available on thirdhand vape residue focuses on the effects of nicotine. Research has failed to examine non-nicotine vapes and the effects of chemicals present in e-liquid and flavouring which may contribute to cell damage.

Thorpe et al. [13] are the only researchers reviewed to date to investigate the impacts of thirdhand non-nicotine vape residue on mouse cells. They reported an insignificant increase in eosinophils, leukocytes, and macrophages but decreased neutrophils in experimental mice, unlike nicotine exposure.

Results reveal that smooth muscle thickness increased in small airways and decreased in large airways, and the layers of epithelial cells decreased in both airways, though not significantly. Like nicotine vapes, increased gene expression contributed to minor increases in small airways’ collagen. Overall, the study revealed a trend in increased pulmonary and central resistance, tissue elastance, and tissue damping in mice exposed to thirdhand non-nicotine vape residue. Notably, exposure to non-nicotine vapes, like nicotine vapes, resulted in statistically significant (*p* < 0.001) alveolar enlargement.

## 4. Discussion

In this study, we set out to investigate the impacts of thirdhand nicotine and non-nicotine vape residue on cells. Statistics have shown that the popularity and use of vapes are increasing [2], suggesting that the amount of thirdhand vape residues are also increasing. However, the exact amount of this is unknown as thirdhand smoke seeps into surfaces and is re-emitted into gases invisible to the plain eye [10,11]. In fact, it is now estimated that a large proportion of secondhand smoke may actually be thirdhand smoke with 5% to 60% of secondhand-related harm attributable to thirdhand exposure [22]. Therefore, investigating the impacts of this on cells was vital.

The findings of this review identified that studies to date only investigated the cellular effects of thirdhand vape residue in mouse models [13,20,21]. However, this was limited, particularly regarding non-nicotine vapes, which were only investigated by Thorpe et al. [13].

In these mice, there was evidence suggesting thirdhand exposure to nicotine vapes has a damaging impact on some cells but not others. This was observed to have an impact on inflammatory cells, decreasing the number of macrophages [13,21] and leukocytes [13]. However, these changes were not significant. The effect on lymphocytes is unclear [20,21]. Contradictions were viewed on the impacts of this exposure in eosinophils and neutrophils [13,21]. Mice exposed to 6 mg of nicotine saw increased neutrophils and decreased eosinophils [21], whereas those exposed to 18 mg of nicotine observed opposite effects [13]. Again, these results demonstrate change but not at a significant level. This minor increase in eosinophils was also viewed in non-nicotine vapes [13]. Case studies have reported vape use to increase eosinophils resulting in acute lung eosinophilic pneumonia [23]. Therefore, the increase in eosinophils viewed in this review may reveal that thirdhand nicotine or non-nicotine vape residue has the potential to be capable of producing similar conditions in mice.

This decrease in inflammatory cells, although non-significant, may suggest that thirdhand nicotine vape residue has a potential anti-inflammatory effect. This finding is consistent with studies demonstrating that nicotine inhibited inflammation and reduced auto-immunity responses in mice with encephalomyelitis [24]. Research in this review by Commodore et al. [21] supports this further, observing a significant increase in Il-13 anti-inflammatory cytokines and a decrease in Il-7 pro-inflammatory cytokines. However, this anti-inflammation may also be detrimental as it increases one’s susceptibility to other serious infections [25].

Conversely, opposing effects in inflammatory cells were viewed in mice after exposure to non-nicotine vapes, with insignificant increases in leukocytes and macrophages but decreased neutrophils [13]. This suggests a possible pro-inflammatory effect which may be attributable to the chemical components of PG, VG, and additive flavours. Research investigating human cell exposure to e-cigarette flavours has similarly viewed increases in pro-inflammatory cytokines significantly impacting lung cells [9], with PG seen to promote asthmatic inflammation and airway hyper-reactivity in asthma models [5]. However, since there is only one reviewed study on thirdhand non-nicotine vapes [13], these results cannot be directly compared and may not be relevant to all cells or models.

Furthermore, the findings of this systematic review revealed that thirdhand nicotine vape residue hyperactivates cells including platelets [20], smooth muscle cells [13], epithelial cells [13], and fibroblastic cells that lay collagen [13]. Increased platelet aggregation and dense granule secretion, enhancing platelet activity, promotes abnormal clumping of platelets to form a haemostatic plug, which increases the chances of chronic cardiac events by occluding blood vessels [26]. Hyperactivation of epithelial, fibroblastic, and smooth muscle cells was observed in mice exposed to thirdhand nicotine and non-nicotine vape residue [13] but at insignificant levels in comparison. Exposure produced smooth muscle and epithelial thickening and collagen deposition in airways [13]. This affects the elasticity and structural integrity of lung airways [27], contributing to reduced central and transpulmonary resistance, tissue damping, and tissue elastance [13] and overall increased respiratory workload [27]. Interestingly, Thorpe et al. [13] discovered that both thirdhand nicotine and non-nicotine vape residue causes alveolar enlargement and degradation comparable to emphysema, a consistent observation found in majority of vape studies in animals [28].

Although no studies reviewed to date focused on the cellular effects of exposure to thirdhand vape residue in human cells, it is possible to compare the results of our review on mice to similar reviews in humans. Olomu et al. [17] and Pozuelos et al. [18] investigated human cells exposed to nicotine at levels typically experienced from thirdhand exposure to vapes. Their findings were consistent with those in this review, observing hyperactivity and increases in human cell number [17] attributable to inhibition of apoptosis pathways [17,18], activation of cell proliferation [17,18] and pathways and hypoxia-inducible factor signalling [18], promoting survival of cells in low oxygen environments. Although these human cell studies only look at the impact of nicotine on cells, this evidence of hyperactivity in possibly even human cells is concerning and may lead to issues beyond the lungs and heart. Notably, one study [29] observed neurobehavioural effects in zebrafish embryos after exposure to flavoured vapes. Gauthier et al. [29] discovered that embryos exposed to flavoured non-nicotine and nicotine vapes had dulled sensory perception and hyperactivity, particularly exacerbated by the flavouring chemicals. This reveals that the combination of chemicals and nicotine in vapes have a dual damaging effect on the body.

### 4.1. Possibilities for Further Research and Action

Overall, the findings of this systematic review call on future research, particularly in investigating the effects of thirdhand vape exposure in human cells and of non-nicotine vapes. This is due to the small number of studies and sample size of these to reduce potential bias. It is evident that this cellular damage extends to broader physiology including tissue and organ damage leading to acute plaque events [26], chronic respiratory conditions [13,23,28], and neurobehavioural conditions among others. These health effects extend beyond the user to those unknowingly exposed to thirdhand smoke. Therefore, findings may guide future studies on therapies and treatments for the various forms of vape-induced cellular damage.

### 4.2. Strengths and Limitations

This systematic review, to the best of our knowledge, is the first to investigate the impacts of thirdhand vape residue from nicotine and non-nicotine vapes on cells. This study follows a strict PRISMA framework for a systematic review to ensure transparency and uses a specific and validated tool for bias assessment of mice models in order to rigorously assess past research on this topic. Assessing a study’s risk of bias is vital for research as it determines the quality, reliability, and validity of the experimental findings. Therefore, the results of studies may be skewed due to potential unclear or high biases. However, in reviews that do not undertake a risk of bias assessment, judgement of the studies included in the review may be flawed due to systematic error, or deviation from the truth in the results or findings drawn from a study. Research reveals that in vivo studies of specific animal disease models, tend to have a low prevalence of reporting methodological details that minimise bias such as randomisation, blinding, reporting of excluded animals and sample size calculation [30]. Studies which fail to mention the use of these controls often report higher intervention efficacy, indicating a high risk of systematic bias rather than a reflection of true effects [30,31]. Therefore, if these details are missing the reliability of the study cannot be determined, as the rigor of a study can only be judged by what is explicitly mentioned. This lack of details is consistent with our findings in this review whereby the included studies also had a great deal of missing information regarding their methodology leading to multiple areas of uncertainty in the reliability and validity of results. This systematic review is also limited particularly due to the very low number of articles available on this particular topic. This may be due to restricting the original search strategy to English published papers only, meaning important papers may have been excluded. Of the articles that are included, heterogeneity of vape type and model particularly is limited. Although this systematic review originally set out to test both animal and human models, due to limited research, all included articles focused on mice models and mostly nicotine vapes. Therefore, the results found are not attributable to all cell types, and the effects of non-nicotine vapes are still debatable. Overall, these results may not be generalisable at this current time, especially for human cells and even non-nicotine vapes, until future evidence is available.

## 5. Conclusions

The findings from this study provide important insight into the partially damaging cellular effects of thirdhand nicotine and non-nicotine vape residue on current mice models. There was significant hyperactivation of cells and changes to the levels of inflammatory cell regulator proteins. However, there was insignificant effects on the inflammatory cells themselves. Although research to date reveals evident physiologic changes to certain cells and tissues in animals, there is no real evidence of likely significant harm in humans. However, considering the increasing popularity of vapes, future research on this unexplored topic, particularly on its effects on humans, is vital for the future of public health.

## Figures and Tables

**Table 1:** Search strategy placed into various databases and the number of articles generated from this.

Bibliographic Search Strategy	Number of Hits
PubMed (Medline):	
(thirdhand*[tw] OR third-hand[tw]) AND (vape*[tw] OR “vaping”[mh] OR vaping[tw] OR e-cigarette*[tw] OR e-cig*[tw] OR ecigarette*[tw] OR ecig*[tw] OR “electronic cigarette*”[tw] OR e-vapor*[tw] OR e-vapour*[tw] OR vapor*[tw] OR vapour*[tw] OR electronic nicotine delivery system*[tw] OR “Electronic Nicotine Delivery Systems”[mh]) AND (“models, animal”[mh] OR “animals, laboratory”[mh] OR Mice[tw] OR mouse[tw] OR murine[tw] OR rats[tw] OR experimental animal*[tw] OR animal stud*[tw] OR laboratory animal*[tw] OR cell* OR cytolog*[tw] OR cytotoxicit*[tw] OR intercell*[tw] OR epithelial*[tw] “cells”[mh] OR “cytology”[mh] OR genotoxic*[tw] OR DNA[tw] OR mitochondria*[tw] OR gene*[tw] OR developmental*[tw] OR Immune function*[tw] OR organs[tw] OR organ system*[tw] OR damag*[tw])	19
Web Of Science	
TS = (thirdhand* OR third-hand) AND TS = (vape* OR vaping OR vaping OR e-cigarette* OR e-cig* OR ecigarette* OR ecig* OR electronic cigarette* OR e-vapor* OR e-vapour* OR vapor* OR vapour* OR electronic nicotine delivery system*) AND TS = (animals, laboratory OR Mice OR mouse OR murine OR rats OR experimental animal* OR animal stud* OR laboratory animal* OR cell* OR cytolog* OR cytotoxicit* OR intercell* OR epithelial* OR cytology OR genotoxic* OR DNA OR mitochondria* OR gene* OR developmental* OR Immune function* OR organ* OR organ system* OR damag*)	45
Scopus	
Article title, abstract, keywords: (thirdhand* OR third-hand) AND (vape* OR vaping OR vaping OR e-cigarette* OR e-cig* OR ecigarette* OR ecig* OR electronic cigarette* OR e-vapor* OR e-vapour* OR vapor* OR vapour*) All fields: (animals, laboratory OR mice OR mouse OR murine OR rats OR experimental animal* OR animal stud* OR laboratory animal* OR cell* OR cytolog* OR cytotoxicit* OR intercell* OR epithelial* OR cytology OR genotoxic* OR dna OR mitochondria* OR gene* OR developmental* OR immune AND function* OR organ* OR organ system* OR damag*)	19
Embase	
((thirdhand* or third-hand) and (vape* or vaping or vaping or e-cigarette* or e-cig* or ecigarette* or ecig* or electronic cigarette* or e-vapor* or e-vapour* or vapor* or vapour* or electronic nicotine delivery system*) and (animals, laboratory or Mice or mouse or murine or rats or experimental animal* or animal stud* or laboratory animal* or cell* or cytolog* or cytotoxicit* or intercell* or epithelial* or cytology or genotoxic* or DNA or mitochondria* or gene* or developmental* or Immune function* or organ* or organ system* or damag*)). mp. (mp = title, abstract, heading word, drug trade name, original title, device manufacturer, drug manufacturer, device trade name, keyword heading word, floating subheading word, candidate term word)	43

**Table 2:** Basic characteristics of included studies.

Authors (Year)	Country	Model and Cell Type	Study Design	Sample Size
Umphres et al. (2024) [20]	USA	Mice model of platelets and inflammatory cells	Controlled experimental	5–8 mice in each group: Thirdhand vape exposure and control
Thorpe et al. (2023) [13]	Australia	Mice model of airway smooth muscle, epithelial and collagen cells, and inflammatory cells	Randomised controlled experimental	10 mice in each group: control, 0 mg nicotine e-vapour, and 18 mg nicotine e-vapour
Commodore et al. (2023) [21]	USA	Mice model of cytokines, chemokines and inflammatory cells	Controlled experimental	7 mice in the experimental group exposed to thirdhand vape smoke and 6 mice in the control group

**Table 3:** Summary of vape exposure and outcome measures of included studies.

Authors (Year)	Cell Type	Type of Vape	Type of Exposure	Duration of Exposure	Outcome Measure
Umphres et al. (2024) [20]	Mice platelets and inflammatory cells.	Nicotine (18 mg).	Cotton fabrics exposed to 400 puffs of e-cigarette vapor per day for 7 days in a vapor inhalation exposure system. Mice lived in cages furnished with vape-infused materials (experimental) or materials exposed to clean air (control).	Mice lived in cages for 4 months.	Blood extracted, platelets activated with thrombin or ADP and analysis of: platelet aggregation and dense granule secretion—measured using aggregometer. Flow cytometric analysis measured through cytometer. Data analysed as: mean and SD, T-test for aggregation and secretion data, one-way-ANOVA for flow cytometry data.
Thorpe et al. (2023) [13]	Mice smooth muscle, epithelial, collagen, and inflammatory cells.	Nicotine (18 mg) and non-nico- tine (0 mg).	100% cotton towels exposed to 20 puffs of e-cigarette vapour (250 mL) with 18 mg or 0 mg nicotine. Mice lived in cages containing nicotine or non-nicotine-infused towel (experimental) or a clean towel (control). Towel replaced daily.	Mice lived in cage for 4 weeks.	BALF extracted, processed, stained, and analysed through imaging. Lungs sectioned, cells stained, mRNA extracted, and analysed through PCR. Data analysed as: mean and SEM, one-way or two-way ANOVA, Tukey’s post hoc tests.
Commodore et al. (2023) [21]	Mice cytokine, chemokines, and inflammatory cells.	Nicotine (6 mg).	100% cotton towel exposed to 1 puff of e-cigarette vapour (70 mL) per minute for 1 h inside a vapor inhalation exposure system. Mice lived in cages containing a vape-infused towel (experimental) or a clean towel (control). Towel replaced after 5 days.	Mice exposed to vape-infused towel for one hour daily for 5 consecutive days over 3 weeks.	Serum and BALF extracted and cell analysed through multiplex protein assay. Data analysed as: mean and SD, one-way ANOVA.

Glossary: adenosine diphosphate (ADP), analysis of variance (ANOVA), bronchoalveolar lavage fluid (BALF), polymerase chain reaction (PCR), standard deviation (SD), standard error of the mean (SEM).

**Table 4:** Risk of bias in animal cell studies assessed using the SYRCLE Risk of Bias tool [15].

Authors (Year)	D1	D2	D3	D4	D5	D6	D7	D8	D9	D10	Overall	
Umphres et al. (2024) [20]	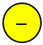	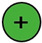	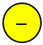	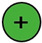	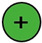	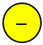	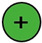	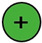	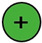	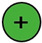	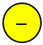	Unclear = but low risk
Thorpe et al. (2023) [13]	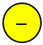	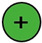	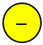	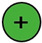	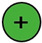	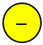	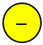	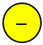	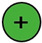	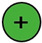	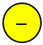	= Unclear
Commodore et al. (2023) [21]	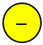	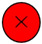	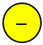	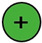	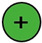	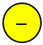	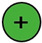	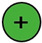	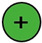	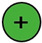	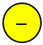	Unclear = but high risk

Key: Low risk (+); unclear risk of bias (−); high risk of bias (✕).

## Data Availability

This review utilised published studies that are available in the public domain.
